# NT-proBNP during and after primary PCI for improved scheduling of early hospital discharge

**DOI:** 10.1007/s12471-016-0935-2

**Published:** 2016-12-09

**Authors:** D. A. A. M. Schellings, A. W. J. van ’t Hof, J. M. ten Berg, A. Elvan, E. Giannitsis, C. Hamm, H. Suryapranata, A. Adiyaman

**Affiliations:** 1 Department of Cardiology, Isala Klinieken, Zwolle, The Netherlands; 2 Department of Cardiology, St. Antonius Hospital, Nieuwegein, The Netherlands; 3 Department of Cardiology, Universitäts Klinik, Heidelberg, Germany; 4 Kerckhoff Klinik, Bad Nauheim, Germany; 5 Department of Cardiology, Radboud University, Nijmegen, The Netherlands; 6 Department of Cardiology, Slingeland Hospital, Doetinchem, The Netherlands

**Keywords:** STEMI, Serial NT-proBNP, PPCI, Zwolle Risk Score, Early discharge, Major adverse cardiac events (MACE)

## Abstract

**Background:**

The Zwolle Risk Score (ZRS) identifies primary percutaneous coronary intervention (PPCI) patients at low mortality risk, eligible for early discharge. Recently, this score was improved by adding baseline NT-proBNP. However, the optimal timepoint for NT-proBNP measurement is unknown.

**Methods:**

PPCI patients in the On-Time 2 study were candidates. The ZRS and NT-proBNP levels on admission, at 18–24 h, at 72–96 h, and the change in NT-proBNP from baseline to 18–24 h (delta NT-proBNP) were determined. We investigated whether addition of the different NT-proBNP measurements to the ZRS improves the prediction of 30-day mortality. Based on cut-off values reflecting zero mortality at 30 d, patients who potentially could be discharged early were identified and occurrence of major adverse cardiac events (MACE) and major bleeding until 10 d was registered.

**Results:**

845 patients were included. On multivariate analyses, NT-proBNP at baseline (HR 2.09, 95% CI 1.59–2.74, *p* < 0.001), at 18–24 h (HR 6.83, 95% CI 2.94–15.84), and at 72–96 h (HR 3.32, 95% CI 1.22–9.06) independently predicted death at 30 d. Addition of NT-proBNP to the ZRS improved prediction of mortality, particularly at 18–24 h (net reclassification index 29%, *p* < 0.0001, integrated discrimination improvement 17%, *p* < 0.0001). Based on ZRS (<2) or NT-proBNP at 18–24 h (<2500 pg/ml) 75% of patients could be targeted for early discharge at 48 h, with expected re-admission rates of 1.2% due to MACE and/or major bleeding.

**Conclusions:**

NT-proBNP at different timepoints improves prognostication of the ZRS. Particularly at 18–24 h post PPCI, the largest group of patients that potentially could be discharged early was identified.

**Electronic supplementary material:**

The online version of this article (doi: 10.1007/s12471-016-0935-2) contains supplementary material, which is available to authorized users.

## Introduction

Primary percutaneous coronary intervention (PPCI) substantially improved outcome in patients with ST-elevation myocardial infarction (STEMI), which has been paralleled by a decrease in hospital length of stay [1–3]. In addition several studies have demonstrated that most low-risk PPCI patients are eligible candidates for early discharge [4–11].

**Fig. 1:**
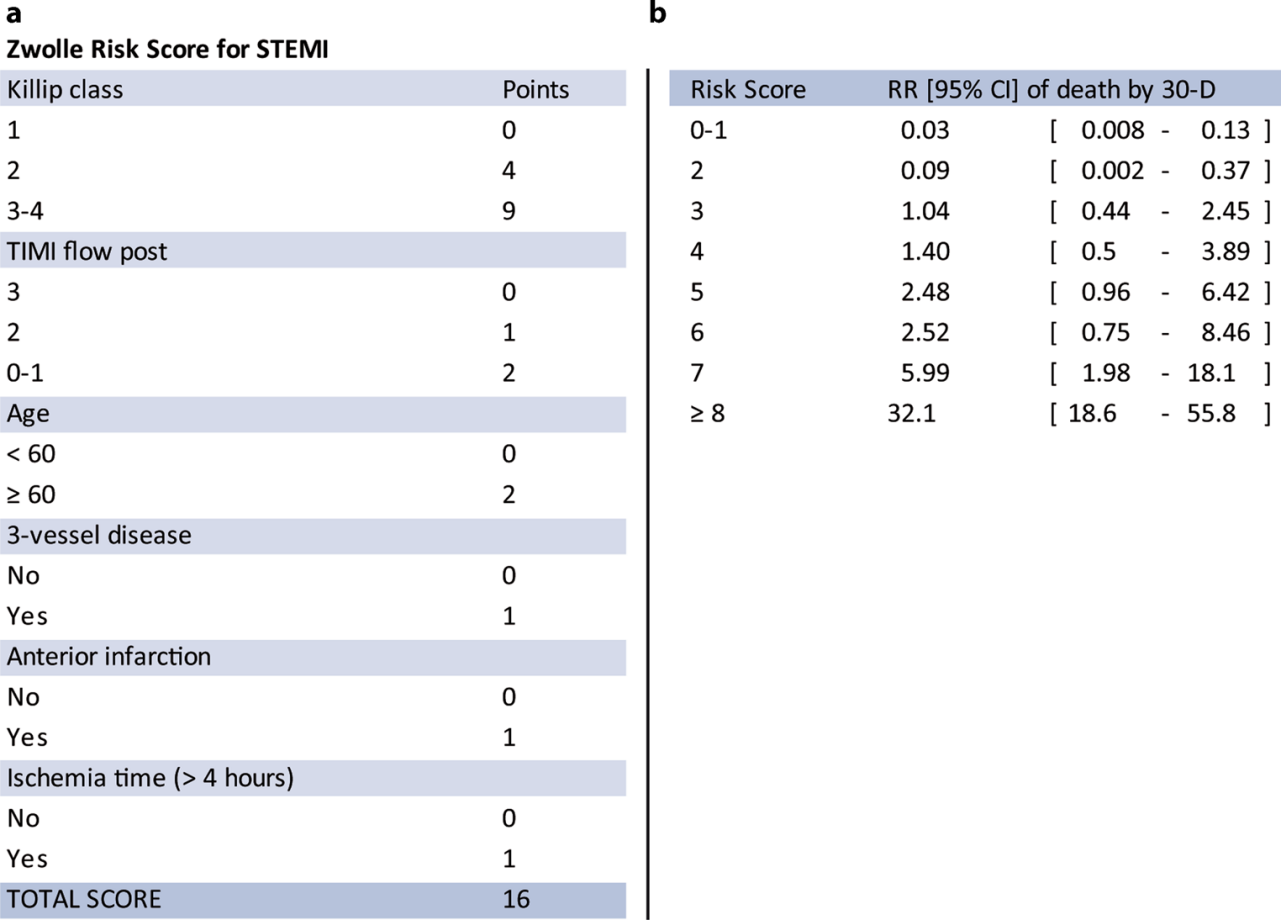
Zwolle Risk Score (**a**) and relative risk (RR) of 30-day mortality for each score (**b**) (*CI* confidence interval, *STEMI* ST-elevation myocardial infarction, *TIMI* Thrombolysis in Myocardial Infarction)

The Zwolle Risk Score (ZRS) is a simple tool designed to identify PPCI patients who can be safely discharged within 72 h, based on their 30-day mortality risk [11]. In patients with a score ≤3 (low risk) the mortality rate was 0.1% at 2 d and 0.2% between 2–10 d and early discharge between 48–72 h could be applied in slightly more than 60% of patients (Fig. 1). Recently, we demonstrated that the predictive accuracy of the ZRS was improved by adding baseline NT-proBNP [12]. In this report we studied the value of NT-proBNP measurement at different timepoints over and beyond the ZRS.

## Methods

### Patients

Our study population consists of patients with the diagnosis of STEMI admitted for PPCI, who were included in the Ongoing Tirofiban in Myocardial Infarction Evaluation (On-TIME) 2 trial [13]. The rationale, design and primary results of this multicentre placebo-controlled, randomised clinical trial have been previously described [13, 14].

Briefly, enrolment was from June 2006 to November 2007. Eligible patients were aged 21–85 years with symptoms of acute myocardial infarction of more than 30 min but less than 24 h and ST-segment elevation of more than 1 mV in two adjacent electrocardiograph (ECG) leads. Exclusion criteria were severe renal dysfunction (glomerular filtration rate <30 ml/min or serum creatinine >200 mmol/l (>2.5 mg/dl)), therapy-resistant cardiogenic shock (systolic blood pressure ≤80 mm Hg for >30 min), persistent severe hypertension (systolic blood pressure >180 mm Hg or diastolic blood pressure >110 mm Hg), or a contraindication to anticoagulation, or increased risk of bleeding. Also, patients with left bundle branch block, pregnant and/or breastfeeding women, and patients with a life expectancy of less than one year were excluded. Written informed consent for participation in both the On-TIME 2 study and future data analysis was obtained from each patient. All local ethics committees involved approved the study protocol.

## Procedures

### Treatment

All patients were planned to undergo PPCI and were initially treated according to the study protocol, randomly assigned to (prehospital) treatment with tirofiban (25 µg/kg bolus and 0.15 µg/kg/min maintenance infusion for 18 h) or placebo. PPCI was performed with standard techniques if the coronary anatomy was suitable for angioplasty. All patients were treated with optimal drug therapy. Final discharge and duration at admission was at the discretion of the treating cardiologist, irrespective of NT-proBNP values or ZRS.

### Measurements

NT-proBNP plasma levels were measured in each patient on admission (after sheath insertion), at 18–24 h and at 72–96 h after PPCI.

NT-proBNP plasma levels were measured by an electrochemiluminiscence immunoassay ‘ECLIA’ on an Elecsys 2010 analyser (Roche, Central Haematology Laboratory, University of Heidelberg, Germany). The ZRS was calculated afterwards in each patient.

### Endpoints

Endpoints were chosen by the ability to preclude early and safe discharge. We took two periods of time: at 48 h (after 2 d) and at 96 h after PPCI (after 3 d as recommended in present guidelines) [1, 2].

The primary endpoint was death from any cause at 30 d after withdrawal of blood from which the NT-proBNP level was determined. Secondary endpoints were non-fatal major adverse cardiac events (MACE) and major bleeding until 10 d after admission. MACE was defined as recurrent myocardial infarction, urgent target vessel revascularisation and malignant cardiac arrhythmias occurring later than 24 h after PPCI, defined as ventricular fibrillation or sustained and non-sustained ventricular tachycardia. Major bleeding was defined as either intracranial bleeding or overt bleeding with a decrease in haemoglobin ≥5 g/dl (≥3.1 mmol/l) or a decrease in haematocrit ≥15%.

## Statistical analysis

Patients were categorised by percentiles of NT-proBNP levels. We assessed the predictive value of the ZRS, serial NT-proBNP levels and the absolute change of NT-proBNP levels from baseline to 18–24 h (delta NT-proBNP) for the primary endpoint.

We computed and plotted receiver operating characteristic (ROC) curves. A total of 74 patients had missing ZRS values and 36 patients did not have baseline NT-proBNP levels. Three patients had missing values of both ZRS and baseline NT-pro BNP. A multiple missing value imputation procedure was applied in which the values of the NT-proBNP levels and the values of the elements of the ZRS were imputed in case they were missing.

We generated 10 datasets and the results of all statistical analyses were pooled across the 10 datasets. Multivariate Cox regression analysis was used to estimate the influence of NT-proBNP and ZRS on 30-day mortality. We adjusted for randomisation, gender, age, renal function (estimated glomerular filtration rate in ml/min), and body mass index in a backward Cox regression with a *p*-value of 0.05 for entry or exit of the variables in the model.

We computed the net reclassification index (NRI) and the integrated discrimination improvement (IDI) to evaluate models with an extra predictor relative to a model with the ZRS alone [15]. The IDI combines the increase in mortality probability for those experiencing an event plus the decrease in mortality probability for those not experiencing an event. The NRI quantifies the net proportion of subjects being correctly reclassified to low or high mortality probabilities when adding a predictor to the model using a cut-off point of 30%.

To analyse models using a linear combination of ZRS and NT-proBNP, we calculated weighted scores for each as follows: (β1 * ZRS) + (β2 * ln NT-proBNP), where β1 and β2 denote β‑coefficients for the ZRS and log NT-proBNP obtained from the multivariate Cox regression model. We used ROC curves to define optimal cut-off values in estimating mortality outcomes. We calculated the pooled areas under the curve (AUC) and 95% confidence intervals by averaging over the 10 AUCs from the imputed datasets using Rubin’s rule [16]. The cut-off values and scores for defining possible early discharge were obtained from the mean scores across the 10 datasets, which gave the highest specificity in combination with a 100% sensitivity for the primary endpoint (e. g. zero mortality 30 d after PPCI). A two-sided *p*-value of ≤0.05 was considered to be statistically significant. The analyses were performed in SPSS version 22 and SAS version 9.3.

## Results

A total of 861 patients underwent PPCI. Complete follow-up was present in 845 patients. Baseline characteristics are shown in Table 1. The mean age was 62 years and 76% were male. Most patients had low Killip class with a post PPCI ejection fraction >40%. Mean hospital stay was 4.89 d.

**Table 1:** Baseline characteristics of the study group

Variable	Total (*n* = 845)	%
Age (years) mean ± SD	62.0 ± 11.5	–
Male gender	642	(76.0)
Current smoking	409	(48.4)
Diabetes mellitus	90	(10.7)
Hypertension	282	(33.4)
Hypercholesterolaemia	222	(26.3)
Family history	340	(40.2)
Prior angina	104	(12.4)
Previous MI	69	(8.2)
Previous PTCA	70	(8.3)
Previous CABG	12	(1.4)
Previous CVA	15	(1.8)
Systolic BP, mean ± SD	130.70 ± 24.21	–
Diastolic BP, mean ± SD	76.57 ± 15.03	–
Killip > I	41	(4.9)
Haemoglobin (mmol/l), mean ± SD	9.49 ± 7.16	–
Troponin (μg/l), mean ± SD	0.37 ± 1.24	–
Zwolle Risk Score, mean ± SD	2.34 ± 2.13	–
Hospital stay (days) mean ± SD	4.89 ± 5.64	–
EF < 30%	41	(6.2)
EF 30–40%	130	(19.8)
EF > 40%	486	(74.0)

All values were denoted as mean ± SD or absolute numbers and their percentages of the total group, where appropriate

*MI* myocardial infarction, *PTCA* percutaneous transluminal coronary angioplasty, *CABG* coronary artery bypass grafting, *CVA* cerebrovascular accident, *BP* blood pressure, *EF* ejection fraction

Baseline NT-proBNP values ranged from 9–33,927 pg/ml (mean 599 ± 1883 pg/ml) with a median of 138 pg/ml. At 18–24 h and 72–96 h, NT-proBNP was determined in 786 and 768 patients respectively, and ranged from 45–20,653 pg/ml (mean 2089 ± 2738 pg/ml, median 1283 pg/ml) and 17–24,119 pg/ml (mean 1618 ± 2556 pg/ml, median 860 pg/ml) respectively.

The primary endpoint was reached in 21 patients. In univariate and multivariate analysis, ZRS and NT-proBNP measured at the different timepoints, but not delta NT-proBNP, were strongly associated with 30-day mortality (Table 2).

**Table 2:** Univariate and multivariate Cox proportional hazards analyses for 30-day mortality

	NT-proBNP baseline (*n* = 845)		NT-proBNP 18–24 h (*n* = 786)		NT-proBNP 72–96 h (*n* = 768)	
Variables	*P*-value	HR (95% CI)	*P*-value	HR (95% CI)	*P*-value	HR (95% CI)
*Univariate analysis*						
Randomisation placebo vs. tirofiban	0.011	4.10 (1.38–12.19)	0.055	4.47 (0.97–20.71)	Cannot be estimated^a^	
Gender (female vs. male)	0.132	1.97 (0.82–4.75)	0.114	2.60 (0.79–8.52)	0.855	0.81 (0.09–7.29)
Age (per year)	<0.001	1.12 (1.06–1.17)	0.006	1.09 (1.03–1.16)	0.179	1.06 (0.97–1.15)
Renal function (eGFR) (per ml/min)	<0.001	0.95 (0.93–0.97)	0.018	0.97 (0.94–0.99)	0.474	0.99 (0.96–1.02)
BMI (per point)	0.602	1.04 (0.91–1.18)	0.318	1.08 (0.93–1.27)	0.964	0.99 (0.76–1.30)
Zwolle Risk Score (per point)	<0.001	1.53 (1.40–1.67)	<0.001	1.59 (1.38–1.84)	<0.001	1.54 (1.24–1.92)
Zwolle Risk Score > 3	<0.001	9.42 (3.49–25.39)	0.008	4.94 (1.51–16.20)	0.246	2.88 (0.48–17.26)
Log NT-proBNP (per point)	<0.001	2.61 (2.00–3.40)	<0.001	9.15 (4.14–20.20)	0.001	4.45 (1.80–10.99)
Delta NT-proBNP (per 100)	–	–	<0.001	1.02 (1.01–1.04)	0.121	0.98 (0.96–1.00)
*Multivariate analysis*						
Log NT-proBNP (per point)	<0.001	2.09 (1.59–2.74)	<0.001	6.83 (2.94–15.84)	0.019	3.32 (1.22–9.06)
Zwolle Risk Score (per point)	<0.001	1.41 (1.27–1.56)	<0.001	1.34 (1.13–1.57)	0.029	1.35 (1.03–1.77)

^a^all 5 deaths were in patients randomised to placebo

*HR* hazard ratio, *eGFR* estimated glomerular filtration rate, *BMI* body mass index

### Predictive value of ZRS and the NT-proBNP measurements on 30-day mortality

The AUCs for ZRS and NT-proBNP at the different timepoints ranged from 0.74–0.87 and 0.87–0.94 respectively. The AUC for delta NT-proBNP was relatively low: 0.74 (95% CI 0.48–0.99). Adding NT-proBNP to the ZRS enlarged the AUCs at all timepoints with the largest area at 18–24 h. In addition, also the highest NRI was acquired by NT-proBNP at 18–24 h. The integrated discrimination improved significantly after addition of NT-proBNP (Table 3).

**Table 3:** Prognostic accuracy of Zwolle Risk Score and NT-proBNP in predicting 30-day mortality at the different timepoints

–	–	AUC	95% CI	NRI (%)	*p* value	IDI (%)	*p* value
Baseline	ZRS	0.87	0.79–0.95	–	–	–	–
	NT-proBNP	0.91	0.86–0.96	–	–	–	–
	ZRS + NT-proBNP	0.94	0.90–0.99	9.82	<0.0001	11.39	<0.0001
18–24 h	ZRS	0.82	0.70–0.94	–	–	–	–
	NT-proBNP	0.94	0.90–0.99	–	–	–	–
	ZRS + NT-proBNP	0.94	0.88–0.99	29.28	<0.0001	17.10	<0.0001
72–96 h	ZRS	0.74	0.54–0.94	–	–	–	–
	NT-proBNP	0.87	0.72–1.00	–	–	–	–
	ZRS + NT-proBNP	0.87	0.71–1.00	21.93	0.0046	7.46	<0.0001

*AUC* area under the curve,* NRI* net reclassification index, *IDI* integrated discrimination improvement

### Cut-off values and potential early discharge

Optimal cut-off values were defined (sensitivity 100%). For NT-proBNP at 18–24 h and at 72–96 h, these were <2500 pg/ml and <1050 pg/ml respectively. Interestingly, the cut-off value of the ZRS had to be <2. Based on the serial test combination, we created decision rules implying that low-risk patients, eligible for early discharge at 48 h after PPCI, had to meet a ZRS <2 or NT-proBNP <2500 pg/ml, withdrawn at 18–24 h after PPCI (group 1). Patients eligible for discharge at 96 h after PPCI had to meet a ZRS <2 or NT-proBNP <1050 pg/ml, withdrawn at 72–96 h after PPCI (group 2).

In group 1, 639 patients met the low-risk criteria and 147 patients were at higher risk (75.6% and 17.3% of the total study population, respectively).

**Fig. 2:**
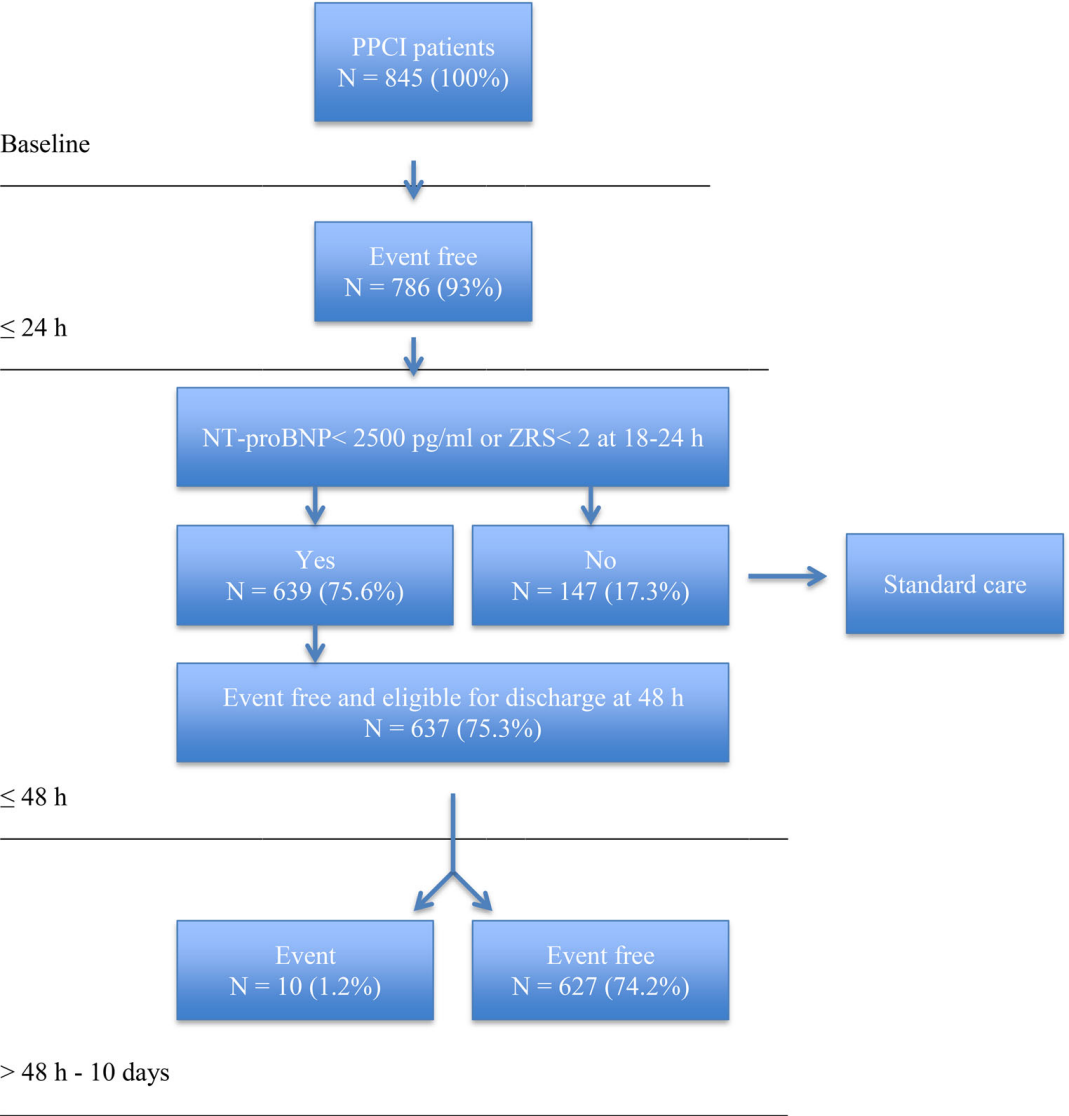
Feasibility of early discharge (after 2 d) in 845 PPCI patients based on ZRS < 2 or NT-proBNP < 2500 pg/ml at 18–24 h

Up to 48 h, MACE occurred significantly more often in higher risk patients than in low-risk patients (7 vs. 2 patients, *p* < 0.001). Major bleeding occurred in 1 patient in both groups (0.2% vs. 0.7%, *p* = 0.34). As a consequence, 637 patients would have been eligible for discharge at 48 h after PPCI. This accounts for 75% of the total study population. Between 48 h and 10 d after PPCI, 10 of these patients (1.2%) experienced adverse events. MACE occurred in 7 patients (0.8%) and major bleeding occurred in 4 patients (0.5%). In 1 patient both events occurred. Therefore, 10 readmissions (1.2%) within 10 d after PPCI would have been expected (Fig. 2).

In group 2, 500 low-risk patients (59%) could be identified versus 268 patients (32%) at higher risk, based on the aforementioned criteria. MACE within 10 d occurred significantly more often in the higher risk group than in the lower risk group (11/268 (4%) vs. 4/500 (0.8%), *p* = 0.003). Major bleeding occurred in 1 patient in the low-risk group (0.1%) and in 5 patients in the higher risk group (0.6%). Hence, readmission would have been necessary in 5 patients. The outcome of all screened patients is presented in Supplementary Fig. 1. The different cut points and outcome are presented in Supplementary Table 1.

## Discussion

Our study demonstrates that NT-proBNP, withdrawn at different timepoints after PPCI, improves prognostication of the established ZRS and consequently may improve the scheduling of early hospital discharge.

Although the clinical outcomes in STEMI patients treated by PPCI have been considerably improved, optimising hospital length of stay in these patients is still challenging.

Recent reports make clear that large groups of patients can be discharged safely at or within 48 h [6, 9, 10], while the present guidelines recommend discharge after a minimum of 3 d [1, 2]. In addition, a lot of international variation in hospital admission duration among low-risk PPCI patients still exists [3, 17–19]. Assuming that the number of low-risk PPCI patients worldwide will increase because of improvement of treatment, there is simultaneously a need for reduction of health care costs. This highlights the necessity for easy tools for risk stratification, in which ideally large proportions of patients could be discharged early. For this purpose, De Luca et al. developed the ZRS. Important variables in this risk model are the success of coronary reperfusion and the occurrence of heart failure. These and other early adverse post PPCI events can also be predicted by NT-proBNP [20–22].

Within this context, NT-proBNP can be considered to be an overlapping variable. The incremental value of adding NT-proBNP to established risk factors may be explained by the fact that biomarker measurement provides more accurate information, without observer variability.

The prognostic significance of the serial changes of plasma NT-proBNP in STEMI patients has previously been studied [23–25]. It was shown that NT-proBNP increases markedly within 24 h. At this timepoint a maximum value is reached after successful reperfusion and then usually decreases, while unsuccessful reperfusion is associated with increased NT-proBNP values at 48–96 h [23, 25].

Consequently, the change in NT-proBNP concentration could also be predictive. In one report, the difference between NT-proBNP at 72 h and baseline NT-proBNP was superior to baseline NT-proBNP, but similar to NT-proBNP at 72 h in predicting short-term adverse cardiac events [25]. Interestingly, in our study the change in NT-proBNP was less predictive and cut-off values were difficult to define, since some patients had negative changes in NT-proBNP (or higher values at baseline). However, measurement at two different timepoints is more laborious and would not be useful within the context of early discharge.

Based on our decision rule at 48 h after PPCI (ZRS < 2 or NT-proBNP < 2500 pg/ml at 18–24 h), a proportion as large as 75% of patients could be discharged early. Therefore NT-proBNP measurement at 18–24 h post PPCI may optimise length of hospital stay in low-risk patients. Furthermore, this risk strategy can also be applied to patients in non-intervention hospitals, since many patients are repatriated in the first 24 h. Especially this group tends to be kept in hospital for a longer period of time [26].

### Limitations

Several limitations of our study have to be noted. Our study lacks a validation cohort to validate the serial model of ZRS and NT-proBNP. Therefore, a future randomised comparison of discharge policies should take place. Although NT-proBNP was measured by protocol in every patient, the ZRS was calculated afterwards, so this may be considered retrospective. Although NT-proBNP has also been related to more cardiovascular risk factors than those used in the regression analysis, more potential confounders could not be included because of the number of events. Finally, the patient population was highly selective since all participants took part in a randomised trial with aforementioned exclusion criteria.

### Conclusion

NT-proBNP at different timepoints improves prognostication of the ZRS. Particularly at 18–24 h post PPCI, the largest group of patients that potentially could be discharged early was identified.

## Caption Electronic Supplementary Material

**Supplementary Fig. 3 **Outcome of all patients screened by the decision rules. Percentages (%) are given with reference to the study population (*n* = 845). Multiple events could occur in one patient

**Supplementary Table 4** Predictive accuracy of ZRS < 2 and NT-proBNP with cut-off values at different timepoints for the identification of PPCI patients eligible for early discharge
