# Supplementary figures and images for: NT-proBNP during and after primary PCI for improved scheduling of early hospital discharge
Source: Neth Heart J. 2016 Dec 9;25(4):243–9. doi: 10.1007/s12471-016-0935-2 (PMC5355383; doi:10.1007/s12471-016-0935-2)

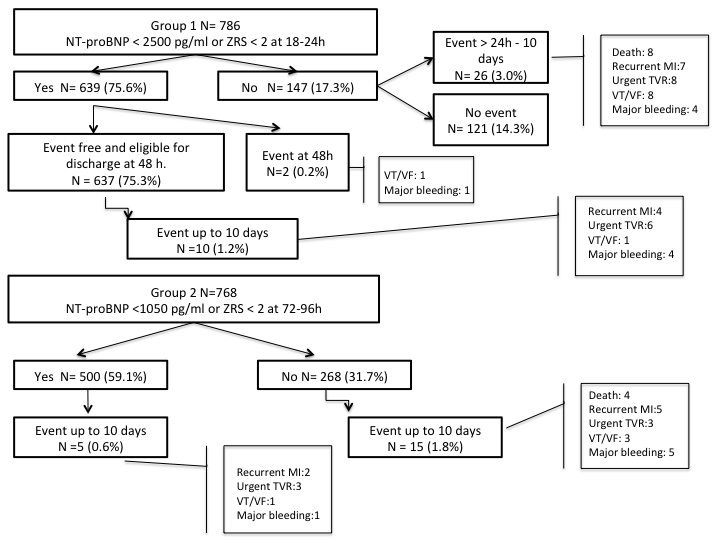

Supplement: Supplementary file 1 — Supplementary Fig. 3 Outcome of all patients screened by the decision rules. Percentages (%) are given with reference to the study population (n = 845). Multiple events could occur in one patient [file 12471_2016_935_MOESM1_ESM.tif]
