# Supplementary material for: NT-proBNP during and after primary PCI for improved scheduling of early hospital discharge
Source: Neth Heart J. 2016 Dec 9;25(4):243–9. doi: 10.1007/s12471-016-0935-2 (PMC5355383; doi:10.1007/s12471-016-0935-2)
Supplement: Supplementary file 2 — Supplementary Table 4 Predictive accuracy of ZRS < 2 and NT-proBNP with cut-off values at different timepoints for the identification of PPCI patients eligible for early discharge [file 12471_2016_935_MOESM2_ESM.docx]

**Supplementary Table 1.**

Predictive accuracy of ZRS <2 and NT-proBNP with cut-off values at different timepoints for the identification of PPCI patients eligible for early discharge, from best to worse (highest specificity, 100% sensitivity)

|  | AUC | Specificity  (95% CI) | % of population early discharge | MACE and bleeding at 10 days among patients eligible for early discharge n (%) |
| --- | --- | --- | --- | --- |
| ZRS <2/ NT-proBNP 18-24 h <2500 pg/ml | 0.94 | 0.82 (0.80-0.85) | 75 | 10 (1.6) |
| NT-proBNP 18-24 h <2500 pg/ml | 0.94 | 0.79 (0.76-0.82) | 72 | 10 (1.6) |
| ZRS<2/ NT-proBNP baseline <200 pg/ml | 0.94 | 0.70 (0.67-0.73) | 64 | 9 (1.7) |
| NT-proBNP baseline <200 pg/ml. | 0.91 | 0.62 (0.58-0.65) | 56 | 7 (1.5) |
| ZRS<2/ NT-proBNP 72-96h <1050 pg/ml | 0.87 | 0.67 (0.64-0.71) | 61 | 5 (1.0) |
| NT-proBNP 72-96 h <1050 pg/ml | 0.87 | 0.56 (0.52-0.59) | 50 | 4 (0.9) |
| ZRS <2 | 0.87 | 0.39 (0.35-0.42) | 36 | 4 (1.3) |

**Appendix**

P-values between specificities

ZRS versus NT proBNP 18-24 h p<0.001

ZRS versus ZRS + NT-proBNP 18-24 h p<0.001

NT-proBNP 18-24h versus ZRS+NT-proBNP 18-24 h p<0.001

ZRS versus NT-proBNP 72-96 h p<0.001

ZRS versus ZRS+ NT-proBNP 72-96 h p<0.001

NT-proBNP 72-96 h versus ZRS+NT-proBNP 72-96 h p<0.001
